# Crystal structure of (*R*)-*N*-benzyl-1-phenylethanaminium (*R*)-4-chloro­mandelate

**DOI:** 10.1107/S1600536814023204

**Published:** 2014-11-05

**Authors:** Yangfeng Peng, Sohrab Rohani, Paul D. Boyle, Quan He

**Affiliations:** a School of Chemical Engineering, East China University of Science and Technology, Shanghai, 200237, People’s Republic of China; b Department of Chemical and Biochemical Engineering, Western University, London, Ontario, N6A 5B9, Canada; c Department of Engineering, Faculty of Agriculture, Dalhousie University, Truro, NovaScotia, B2N 5E3, Canada

**Keywords:** Crystal structure, 4-chloro­mandelate, diastereomeric salt, resolution, absolute structure, resonant scattering, hydrogen bonding, C—H⋯π inter­actions, Cl⋯Cl inter­action

## Abstract

The absolute configuration of the title mol­ecular salt, C_15_H_18_N^+^·C_8_H_6_ClO_3_^−^, has been confirmed by resonant scattering. In the (*R*)-*N*-benzyl-1-phenyl-ethyl­ammonium cation, the phenyl rings are inclined to one another by 44.65 (7)°. In the crystal, the (*R*)-4-chloro­mandelate anions are linked *via* O—H⋯O hydrogen bonds and bridged by N—H⋯O hydrogen bonds involving the cations, forming chains along [010]. There are C—H⋯O hydrogen bonds present within the chains, which are linked *via* C—H⋯π inter­actions and a short Cl⋯Cl inter­action [3.193 (1) Å] forming a three-dimensional framework. The structure was refined as a two-component inversion twin giving a Flack parameter of 0.05 (4).

## Related literature   

For the resolution of chlorine-substituted mandelic acids, see: He, Gomaa *et al.* (2010 ▶); He, Peng *et al.* (2010 ▶); Peng *et al.* (2012 ▶).
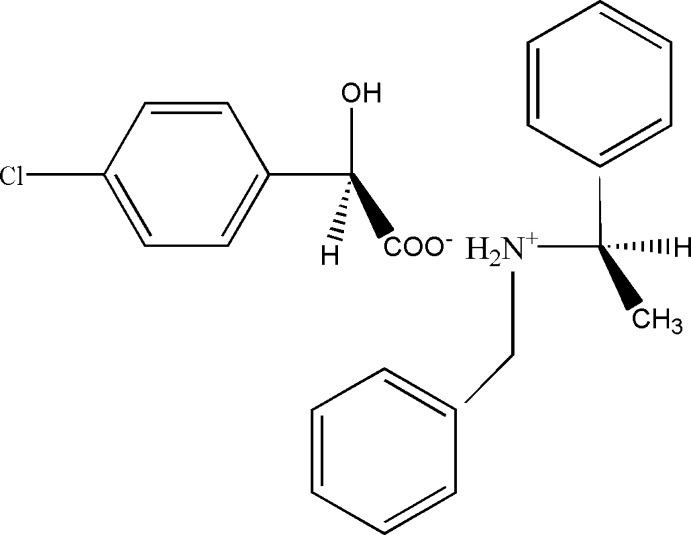


## Experimental   

### Crystal data  

C_15_H_18_N^+^·C_8_H_6_ClO_3_^−^
*M* *_r_* = 397.88
Monoclinic, 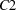

*a* = 17.783 (5) Å
*b* = 9.6993 (19) Å
*c* = 12.796 (3) Å
β = 107.868 (10)°
*V* = 2100.6 (8) Å^3^
*Z* = 4
Mo *K*α radiation
μ = 0.21 mm^−1^
*T* = 110 K
0.56 × 0.13 × 0.12 mm

### Data collection  

Bruker APEXII diffractometer
Absorption correction: multi-scan (*SADABS*; Sheldrick, 1996 ▶) *T*_min_ = 0.685, *T*_max_ = 0.747
34940 measured reflections
7574 independent reflections
6778 reflections with *I* > 2σ(*I*)
*R*_int_ = 0.031

### Refinement  

*R*[*F* ^2^ > 2σ(*F* ^2^)] = 0.036
*wR*(*F* ^2^) = 0.092
*S* = 1.04
7574 reflections
350 parameters
1 restraint
All H-atom parameters refined
Δρ_max_ = 0.34 e Å^−3^
Δρ_min_ = −0.45 e Å^−3^
Absolute structure: Refined as an inversion twin.
Absolute structure parameter: 0.05 (4)

### 

Data collection: *APEX2* (Bruker, 2009 ▶); cell refinement: *SAINT* (Bruker, 2009 ▶); data reduction: *SAINT*); program(s) used to solve structure: *SHELXT* (Sheldrick, 2008 ▶); program(s) used to refine structure: *SHELXL2014* (Sheldrick, 2008 ▶); molecular graphics: *PLATON* (Spek, 2009 ▶); software used to prepare material for publication: *SHELXL2014*, *PLATON* and *publCIF* (Westrip, 2010 ▶).

## Supplementary Material

Crystal structure: contains datablock(s) I, Global. DOI: 10.1107/S1600536814023204/su5008sup1.cif

Structure factors: contains datablock(s) I. DOI: 10.1107/S1600536814023204/su5008Isup2.hkl

**Fig. 1.**
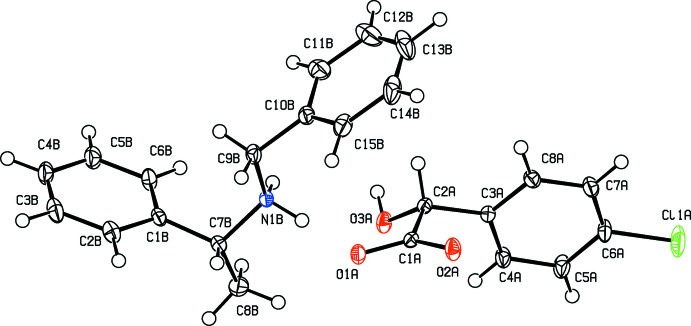
A view of the molecular structure of the title salt, with atom labelling. Displacement ellipsoids are drawn at the 50% probability level.

**Fig. 2.**
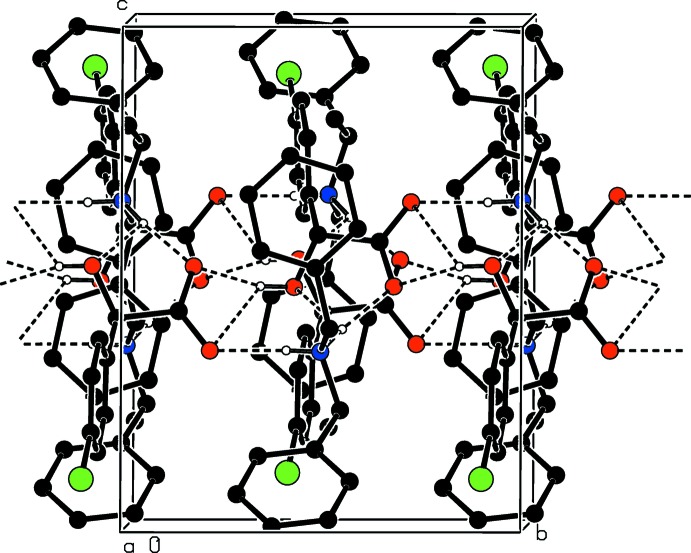
A view along the a axis of the crystal packing of the title molecular salt. The O-H···O and N-H···O hydrogen bonds are shown as dashed lines (see Table 1 for details; C-bound H atoms have been omitted for clarity).

. DOI: 10.1107/S1600536814023204/su5008fig1.tif
A view of the mol­ecular structure of the title salt, with atom labelling. Displacement ellipsoids are drawn at the 50% probability level.

a . DOI: 10.1107/S1600536814023204/su5008fig2.tif
A view along the *a* axis of the crystal packing of the title mol­ecular salt. The O-H⋯O and N-H⋯O hydrogen bonds are shown as dashed lines (see Table 1 for details; C-bound H atoms have been omitted for clarity).

CCDC reference: 1030316

Additional supporting information:  crystallographic information; 3D view; checkCIF report

## Figures and Tables

**Table 1:** Hydrogen-bond geometry (, ) *Cg*1 and *Cg*2 are the centroids of rings C1*B*C6*B* and C10*B*C15*B*, respectively.

*D*H*A*	*D*H	H*A*	*D* *A*	*D*H*A*
O3*A*H3*A*O1*A* ^i^	0.87(2)	1.84(2)	2.6878(15)	164.9(17)
O3*A*H3*A*O2*A* ^i^	0.87(2)	2.52(2)	3.1629(14)	130.6(16)
N1*B*H1*BA*O2*A* ^i^	0.85(2)	1.90(2)	2.7457(16)	176.1(16)
N1*B*H1*BB*O1*A*	0.98(2)	1.78(2)	2.7337(15)	163.7(18)
N1*B*H1*BB*O3*A*	0.98(2)	2.42(2)	3.0019(14)	117.4(15)
C6*B*H6*B*O2*A* ^i^	0.92(2)	2.39(2)	3.2275(19)	152.1(15)
C2*A*H2*A* *Cg*2	1.00(2)	2.827(19)	3.7029(18)	146.7(14)
C9*B*H9*B*2*Cg*2^ii^	0.97(2)	2.69(2)	3.4243(17)	146.7(14)
C7*A*H7*A* *Cg*1^iii^	0.99(2)	2.753(19)	3.6111(18)	145.5(15)

Symmetry codes: (i) 
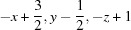
; (ii) 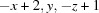; (iii) 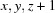.

## supplementary crystallographic information

### S1. Chemical context

In our on-going research work on the resolution of chlorine-substituted mandelic acids with optically active phenyl-ethyl-amine­(PEA) and it was found that that PEA was an excellent resolving agent for the resolution of racemic 4-chloro-mandelic acid (He, Gomaa *et al.*, 2010; He, Peng *et al.*, 2010). However, it failed to resolve racemic 2-chloro-mandelic acid. A benzyl functional group was introduced in PEA, leading to a new resolving agent, N-benzyl-phenyl-ethyl-amine­(BPA), which demonstrated a high resolution efficiency in the resolution of 2-chloro-mandelic acid (Peng *et al.*, 2012). In order to obtain insight into the enhanced chiral discrimination ability of BPA, the resolution of 4-chloro-mandelic acid with BPA has been investigated, and the single crystal structure of the resulting less soluble diastereomeric title salt, is reported on herein.

The title compound consists of an ion pair; an amine cation and a carboxyl­ate anion (Fig. 1). The absolute stereochemistry of each ion has been confirmed by resonant scattering.

In the crystal, the (R)-4-chloro-mandelate anions are linked via O—H···O hydrogen bonds and bridged by N—H···O hydrogen bonds involving the cations forming chains along [010], see Table 1 and Fig. 2. There are C—H···O hydrogen bonds present within the chains which are linked via C—H···π inter­actions (Table 1), and a short Cl1···Cl1^i^ inter­action [3.193 (1) Å; symmetry code: (i) -x + 1, y, -z + 2], forming a three-dimensional framework.

### S2. Refinement details

All of the hydrogen atoms were located in difference Fourier maps and freely refined. The structure was refined as a 2-component inversion twin giving a Flack parameter of 0.05 (4).

### Crystal data

**Table:**

C_15_H_18_N^+^·C_8_H_6_ClO_3_^−^	*F*(000) = 840
*M**_r_* = 397.88	*D*_x_ = 1.258 Mg m^−^^3^
Monoclinic, *C*2	Mo *K*α radiation, λ = 0.71073 Å
*a* = 17.783 (5) Å	Cell parameters from 9969 reflections
*b* = 9.6993 (19) Å	θ = 2.5–35.8°
*c* = 12.796 (3) Å	µ = 0.21 mm^−^^1^
β = 107.868 (10)°	*T* = 110 K
*V* = 2100.6 (8) Å^3^	Prism, colourless
*Z* = 4	0.56 × 0.13 × 0.12 mm

### Data collection

**Table:**

Bruker APEXII diffractometer	6778 reflections with *I* > 2σ(*I*)
Radiation source: sealed tube	*R*_int_ = 0.031
phi and ω scans	θ_max_ = 37.0°, θ_min_ = 3.8°
Absorption correction: multi-scan (*SADABS*; Sheldrick, 1996)	*h* = −27→29
*T*_min_ = 0.685, *T*_max_ = 0.747	*k* = −10→16
34940 measured reflections	*l* = −21→19
7574 independent reflections	

### Refinement

**Table:**

Refinement on *F*^2^	Secondary atom site location: difference Fourier map
Least-squares matrix: full	Hydrogen site location: difference Fourier map
*R*[*F*^2^ > 2σ(*F*^2^)] = 0.036	All H-atom parameters refined
*wR*(*F*^2^) = 0.092	*w* = 1/[σ^2^(*F*_o_^2^) + (0.0583*P*)^2^ + 0.0666*P*] where *P* = (*F*_o_^2^ + 2*F*_c_^2^)/3
*S* = 1.04	(Δ/σ)_max_ < 0.001
7574 reflections	Δρ_max_ = 0.34 e Å^−^^3^
350 parameters	Δρ_min_ = −0.45 e Å^−^^3^
1 restraint	Absolute structure: Refined as an inversion twin.
Primary atom site location: dual	Absolute structure parameter: 0.05 (4)

### Special details

**Table:**

Geometry. All e.s.d.'s (except the e.s.d. in the dihedral angle between two l.s. planes) are estimated using the full covariance matrix. The cell e.s.d.'s are taken into account individually in the estimation of e.s.d.'s in distances, angles and torsion angles; correlations between e.s.d.'s in cell parameters are only used when they are defined by crystal symmetry. An approximate (isotropic) treatment of cell e.s.d.'s is used for estimating e.s.d.'s involving l.s. planes.
Refinement. Refined as a 2-component inversion twin.

### Fractional atomic coordinates and isotropic or equivalent isotropic displacement parameters (Å^2^)

**Table:**

	*x*	*y*	*z*	*U*_iso_*/*U*_eq_	
O1A	0.74654 (6)	0.67270 (10)	0.48017 (7)	0.01801 (17)	
O2A	0.73497 (6)	0.71450 (10)	0.64630 (8)	0.02089 (19)	
C1A	0.73400 (7)	0.63607 (12)	0.56813 (9)	0.0140 (2)	
C2A	0.71982 (7)	0.48180 (12)	0.58184 (9)	0.01356 (19)	
H2A	0.7731 (10)	0.439 (2)	0.6135 (14)	0.018 (4)*	
O3A	0.68420 (5)	0.42149 (10)	0.47752 (7)	0.01833 (17)	
H3A	0.7016 (11)	0.338 (2)	0.4792 (15)	0.019 (4)*	
C3A	0.67231 (7)	0.45775 (12)	0.65978 (9)	0.0135 (2)	
C4A	0.59064 (7)	0.44317 (16)	0.62065 (10)	0.0211 (3)	
H4A	0.5657 (12)	0.446 (2)	0.5482 (16)	0.027 (5)*	
C5A	0.54612 (7)	0.42553 (19)	0.69271 (10)	0.0250 (3)	
H5A	0.4896 (14)	0.413 (3)	0.6653 (18)	0.045 (6)*	
C6A	0.58539 (8)	0.42250 (16)	0.80456 (10)	0.0210 (2)	
Cl1A	0.53108 (2)	0.40049 (6)	0.89547 (3)	0.03874 (12)	
C7A	0.66664 (8)	0.43605 (14)	0.84569 (10)	0.0195 (2)	
H7A	0.6948 (11)	0.435 (2)	0.9253 (15)	0.024 (5)*	
C8A	0.70965 (7)	0.45356 (14)	0.77258 (10)	0.0168 (2)	
H8A	0.7667 (11)	0.4584 (18)	0.7988 (14)	0.015 (4)*	
C1B	0.80429 (7)	0.47938 (13)	0.15898 (9)	0.0159 (2)	
C2B	0.83482 (8)	0.56797 (15)	0.09643 (10)	0.0213 (2)	
H2B	0.8295 (15)	0.673 (3)	0.105 (2)	0.043 (6)*	
C3B	0.87017 (9)	0.51600 (17)	0.02130 (11)	0.0248 (3)	
H3B	0.8938 (12)	0.582 (2)	−0.0200 (16)	0.032 (5)*	
C4B	0.87563 (8)	0.37495 (18)	0.00863 (10)	0.0244 (3)	
H4B	0.9019 (12)	0.345 (2)	−0.0409 (17)	0.031 (5)*	
C5B	0.84581 (9)	0.28592 (16)	0.07139 (11)	0.0239 (3)	
H5B	0.8480 (13)	0.188 (3)	0.0656 (18)	0.036 (6)*	
C6B	0.80992 (9)	0.33748 (15)	0.14612 (10)	0.0203 (2)	
H6B	0.7919 (10)	0.277 (2)	0.1883 (14)	0.016 (4)*	
C7B	0.76252 (7)	0.54065 (14)	0.23543 (9)	0.0163 (2)	
H7B	0.7728 (11)	0.637 (2)	0.2405 (15)	0.020 (4)*	
C8B	0.67419 (8)	0.50930 (18)	0.19966 (11)	0.0254 (3)	
H8B1	0.6469 (13)	0.540 (3)	0.1221 (18)	0.041 (6)*	
H8B2	0.6493 (12)	0.548 (3)	0.2523 (17)	0.035 (5)*	
H8B3	0.6662 (11)	0.412 (3)	0.1986 (15)	0.028 (5)*	
N1B	0.79666 (6)	0.49143 (11)	0.35198 (8)	0.01298 (17)	
H1BA	0.7855 (9)	0.406 (2)	0.3544 (12)	0.012 (4)*	
H1BB	0.7704 (11)	0.545 (2)	0.3959 (16)	0.023 (4)*	
C9B	0.88367 (7)	0.51675 (15)	0.39986 (10)	0.0177 (2)	
H9B1	0.8911 (11)	0.614 (2)	0.3831 (15)	0.021 (4)*	
H9B2	0.9115 (11)	0.463 (2)	0.3600 (15)	0.020 (4)*	
C10B	0.91101 (7)	0.48164 (15)	0.52067 (10)	0.0179 (2)	
C11B	0.92328 (8)	0.34526 (18)	0.55489 (12)	0.0257 (3)	
H11B	0.9173 (12)	0.277 (2)	0.5014 (16)	0.024 (5)*	
C12B	0.94738 (9)	0.3151 (2)	0.66696 (15)	0.0375 (4)	
H12B	0.9511 (15)	0.221 (3)	0.679 (2)	0.048 (7)*	
C13B	0.95961 (9)	0.4192 (3)	0.74345 (12)	0.0444 (5)	
H13B	0.9770 (14)	0.393 (3)	0.8221 (19)	0.049 (6)*	
C14B	0.94846 (9)	0.5551 (3)	0.71019 (12)	0.0378 (4)	
H14B	0.9609 (15)	0.631 (3)	0.766 (2)	0.044 (6)*	
C15B	0.92392 (8)	0.58726 (18)	0.59849 (11)	0.0253 (3)	
H15B	0.9134 (11)	0.688 (2)	0.5689 (16)	0.024 (5)*	

### Atomic displacement parameters (Å^2^)

**Table:**

	*U*^11^	*U*^22^	*U*^33^	*U*^12^	*U*^13^	*U*^23^
O1A	0.0260 (4)	0.0155 (4)	0.0164 (4)	0.0021 (3)	0.0121 (3)	0.0014 (3)
O2A	0.0338 (5)	0.0152 (4)	0.0178 (4)	0.0002 (4)	0.0141 (4)	−0.0018 (3)
C1A	0.0155 (5)	0.0135 (5)	0.0146 (4)	0.0028 (4)	0.0069 (4)	0.0006 (4)
C2A	0.0144 (4)	0.0135 (5)	0.0140 (4)	0.0014 (4)	0.0061 (4)	−0.0005 (4)
O3A	0.0245 (4)	0.0179 (4)	0.0151 (4)	−0.0011 (4)	0.0098 (3)	−0.0048 (3)
C3A	0.0141 (4)	0.0143 (5)	0.0127 (4)	0.0003 (4)	0.0052 (4)	0.0001 (3)
C4A	0.0142 (5)	0.0374 (8)	0.0118 (5)	−0.0011 (5)	0.0038 (4)	−0.0010 (4)
C5A	0.0145 (5)	0.0447 (9)	0.0169 (5)	−0.0031 (6)	0.0067 (4)	−0.0027 (5)
C6A	0.0234 (5)	0.0285 (7)	0.0152 (5)	−0.0023 (5)	0.0118 (4)	−0.0008 (4)
Cl1A	0.0331 (2)	0.0675 (3)	0.0237 (2)	−0.0058 (2)	0.0206 (1)	−0.0011 (2)
C7A	0.0226 (5)	0.0240 (6)	0.0125 (4)	−0.0005 (5)	0.0061 (4)	0.0008 (4)
C8A	0.0153 (5)	0.0210 (6)	0.0136 (4)	−0.0008 (4)	0.0036 (4)	0.0017 (4)
C1B	0.0181 (5)	0.0191 (5)	0.0104 (4)	−0.0002 (4)	0.0041 (4)	0.0016 (4)
C2B	0.0243 (6)	0.0237 (6)	0.0162 (5)	−0.0040 (5)	0.0068 (4)	0.0021 (4)
C3B	0.0253 (6)	0.0348 (8)	0.0157 (5)	−0.0062 (6)	0.0086 (5)	0.0028 (5)
C4B	0.0223 (6)	0.0390 (8)	0.0135 (5)	0.0008 (6)	0.0078 (4)	−0.0022 (5)
C5B	0.0327 (7)	0.0243 (7)	0.0173 (5)	0.0028 (5)	0.0116 (5)	−0.0012 (4)
C6B	0.0278 (6)	0.0210 (6)	0.0152 (5)	−0.0012 (5)	0.0110 (5)	0.0003 (4)
C7B	0.0193 (5)	0.0177 (5)	0.0122 (4)	0.0023 (4)	0.0051 (4)	0.0024 (4)
C8B	0.0173 (5)	0.0390 (9)	0.0185 (5)	0.0047 (6)	0.0035 (4)	0.0002 (5)
N1B	0.0140 (4)	0.0145 (4)	0.0111 (4)	−0.0002 (3)	0.0049 (3)	−0.0002 (3)
C9B	0.0140 (5)	0.0261 (6)	0.0139 (4)	−0.0032 (4)	0.0056 (4)	0.0006 (4)
C10B	0.0120 (4)	0.0278 (6)	0.0140 (5)	−0.0008 (4)	0.0040 (4)	0.0003 (4)
C11B	0.0166 (5)	0.0327 (7)	0.0264 (6)	−0.0015 (5)	0.0046 (5)	0.0071 (5)
C12B	0.0200 (6)	0.0543 (12)	0.0353 (8)	−0.0022 (7)	0.0041 (6)	0.0247 (8)
C13B	0.0196 (6)	0.0945 (17)	0.0170 (6)	−0.0065 (9)	0.0024 (5)	0.0128 (8)
C14B	0.0206 (6)	0.0767 (14)	0.0157 (6)	−0.0034 (7)	0.0051 (5)	−0.0103 (7)
C15B	0.0164 (5)	0.0407 (8)	0.0183 (5)	−0.0015 (5)	0.0047 (4)	−0.0079 (5)

### Geometric parameters (Å, º)

**Table:**

O1A—C1A	1.2636 (14)	C5B—C6B	1.395 (2)
O2A—C1A	1.2525 (15)	C5B—H5B	0.95 (2)
C1A—C2A	1.5364 (17)	C6B—H6B	0.921 (19)
C2A—O3A	1.4164 (14)	C7B—N1B	1.5051 (15)
C2A—C3A	1.5100 (16)	C7B—C8B	1.5258 (19)
C2A—H2A	1.001 (17)	C7B—H7B	0.95 (2)
O3A—H3A	0.87 (2)	C8B—H8B1	1.01 (2)
C3A—C4A	1.3905 (16)	C8B—H8B2	0.99 (2)
C3A—C8A	1.3920 (16)	C8B—H8B3	0.95 (3)
C4A—C5A	1.3979 (18)	N1B—C9B	1.4994 (16)
C4A—H4A	0.897 (19)	N1B—H1BA	0.85 (2)
C5A—C6A	1.3869 (17)	N1B—H1BB	0.98 (2)
C5A—H5A	0.97 (2)	C9B—C10B	1.5103 (17)
C6A—C7A	1.3839 (18)	C9B—H9B1	0.98 (2)
C6A—Cl1A	1.7381 (13)	C9B—H9B2	0.968 (19)
C7A—C8A	1.3886 (17)	C10B—C11B	1.389 (2)
C7A—H7A	0.988 (19)	C10B—C15B	1.398 (2)
C8A—H8A	0.967 (18)	C11B—C12B	1.396 (2)
C1B—C2B	1.3931 (18)	C11B—H11B	0.94 (2)
C1B—C6B	1.3934 (19)	C12B—C13B	1.376 (4)
C1B—C7B	1.5191 (18)	C12B—H12B	0.93 (3)
C2B—C3B	1.394 (2)	C13B—C14B	1.381 (4)
C2B—H2B	1.03 (3)	C13B—H13B	0.99 (2)
C3B—C4B	1.385 (2)	C14B—C15B	1.396 (2)
C3B—H3B	1.00 (2)	C14B—H14B	1.00 (3)
C4B—C5B	1.390 (2)	C15B—H15B	1.04 (2)
C4B—H4B	0.94 (2)		
			
O2A—C1A—O1A	125.28 (12)	C1B—C6B—H6B	120.9 (12)
O2A—C1A—C2A	117.57 (10)	C5B—C6B—H6B	119.1 (12)
O1A—C1A—C2A	117.10 (10)	N1B—C7B—C1B	112.69 (10)
O3A—C2A—C3A	112.31 (10)	N1B—C7B—C8B	107.40 (11)
O3A—C2A—C1A	109.65 (10)	C1B—C7B—C8B	113.00 (11)
C3A—C2A—C1A	111.76 (10)	N1B—C7B—H7B	103.5 (11)
O3A—C2A—H2A	107.7 (10)	C1B—C7B—H7B	107.9 (11)
C3A—C2A—H2A	108.7 (10)	C8B—C7B—H7B	111.9 (11)
C1A—C2A—H2A	106.5 (11)	C7B—C8B—H8B1	112.1 (13)
C2A—O3A—H3A	108.1 (12)	C7B—C8B—H8B2	110.9 (12)
C4A—C3A—C8A	118.93 (11)	H8B1—C8B—H8B2	112.3 (19)
C4A—C3A—C2A	120.80 (10)	C7B—C8B—H8B3	109.7 (11)
C8A—C3A—C2A	120.24 (10)	H8B1—C8B—H8B3	104.9 (18)
C3A—C4A—C5A	121.01 (11)	H8B2—C8B—H8B3	106.6 (18)
C3A—C4A—H4A	119.9 (13)	C9B—N1B—C7B	113.88 (10)
C5A—C4A—H4A	119.1 (13)	C9B—N1B—H1BA	111.5 (11)
C6A—C5A—C4A	118.39 (11)	C7B—N1B—H1BA	108.4 (10)
C6A—C5A—H5A	120.7 (13)	C9B—N1B—H1BB	107.0 (11)
C4A—C5A—H5A	120.9 (13)	C7B—N1B—H1BB	106.3 (11)
C7A—C6A—C5A	121.79 (11)	H1BA—N1B—H1BB	109.5 (16)
C7A—C6A—Cl1A	119.13 (9)	N1B—C9B—C10B	110.43 (10)
C5A—C6A—Cl1A	119.08 (10)	N1B—C9B—H9B1	105.0 (11)
C6A—C7A—C8A	118.82 (11)	C10B—C9B—H9B1	114.6 (11)
C6A—C7A—H7A	122.0 (11)	N1B—C9B—H9B2	109.1 (11)
C8A—C7A—H7A	119.1 (11)	C10B—C9B—H9B2	111.2 (11)
C7A—C8A—C3A	121.06 (11)	H9B1—C9B—H9B2	106.3 (16)
C7A—C8A—H8A	120.5 (11)	C11B—C10B—C15B	119.85 (13)
C3A—C8A—H8A	118.4 (11)	C11B—C10B—C9B	120.48 (12)
C2B—C1B—C6B	119.12 (13)	C15B—C10B—C9B	119.67 (13)
C2B—C1B—C7B	118.85 (12)	C10B—C11B—C12B	119.48 (17)
C6B—C1B—C7B	121.98 (12)	C10B—C11B—H11B	118.3 (13)
C1B—C2B—C3B	120.71 (14)	C12B—C11B—H11B	122.2 (13)
C1B—C2B—H2B	119.1 (14)	C13B—C12B—C11B	120.58 (19)
C3B—C2B—H2B	120.2 (14)	C13B—C12B—H12B	127.9 (16)
C4B—C3B—C2B	120.04 (13)	C11B—C12B—H12B	111.4 (16)
C4B—C3B—H3B	120.9 (12)	C12B—C13B—C14B	120.31 (14)
C2B—C3B—H3B	119.0 (12)	C12B—C13B—H13B	117.7 (18)
C3B—C4B—C5B	119.55 (13)	C14B—C13B—H13B	122.0 (18)
C3B—C4B—H4B	117.0 (13)	C13B—C14B—C15B	119.94 (17)
C5B—C4B—H4B	123.4 (13)	C13B—C14B—H14B	120.0 (14)
C4B—C5B—C6B	120.60 (14)	C15B—C14B—H14B	120.0 (14)
C4B—C5B—H5B	122.7 (13)	C14B—C15B—C10B	119.82 (17)
C6B—C5B—H5B	116.7 (13)	C14B—C15B—H15B	123.1 (11)
C1B—C6B—C5B	119.97 (13)	C10B—C15B—H15B	117.0 (11)
			
O2A—C1A—C2A—O3A	152.48 (11)	C3B—C4B—C5B—C6B	0.6 (2)
O1A—C1A—C2A—O3A	−29.96 (14)	C2B—C1B—C6B—C5B	0.0 (2)
O2A—C1A—C2A—C3A	27.28 (15)	C7B—C1B—C6B—C5B	177.19 (12)
O1A—C1A—C2A—C3A	−155.16 (10)	C4B—C5B—C6B—C1B	−0.5 (2)
O3A—C2A—C3A—C4A	−29.54 (16)	C2B—C1B—C7B—N1B	−125.48 (12)
C1A—C2A—C3A—C4A	94.18 (14)	C6B—C1B—C7B—N1B	57.34 (16)
O3A—C2A—C3A—C8A	152.20 (11)	C2B—C1B—C7B—C8B	112.54 (14)
C1A—C2A—C3A—C8A	−84.08 (14)	C6B—C1B—C7B—C8B	−64.64 (16)
C8A—C3A—C4A—C5A	0.5 (2)	C1B—C7B—N1B—C9B	55.64 (14)
C2A—C3A—C4A—C5A	−177.82 (14)	C8B—C7B—N1B—C9B	−179.28 (11)
C3A—C4A—C5A—C6A	−0.2 (2)	C7B—N1B—C9B—C10B	172.64 (11)
C4A—C5A—C6A—C7A	−0.2 (2)	N1B—C9B—C10B—C11B	78.45 (15)
C4A—C5A—C6A—Cl1A	−179.94 (13)	N1B—C9B—C10B—C15B	−101.46 (14)
C5A—C6A—C7A—C8A	0.2 (2)	C15B—C10B—C11B—C12B	0.9 (2)
Cl1A—C6A—C7A—C8A	179.98 (11)	C9B—C10B—C11B—C12B	−179.01 (12)
C6A—C7A—C8A—C3A	0.1 (2)	C10B—C11B—C12B—C13B	−0.5 (2)
C4A—C3A—C8A—C7A	−0.41 (19)	C11B—C12B—C13B—C14B	−0.3 (2)
C2A—C3A—C8A—C7A	177.88 (12)	C12B—C13B—C14B—C15B	0.6 (2)
C6B—C1B—C2B—C3B	0.4 (2)	C13B—C14B—C15B—C10B	−0.3 (2)
C7B—C1B—C2B—C3B	−176.84 (12)	C11B—C10B—C15B—C14B	−0.5 (2)
C1B—C2B—C3B—C4B	−0.4 (2)	C9B—C10B—C15B—C14B	179.39 (13)
C2B—C3B—C4B—C5B	−0.1 (2)		

### Hydrogen-bond geometry (Å, º)

Cg1 and Cg2 are the centroids of rings C1B–C6B and C10B–C15B, respectively.

**Table:**

*D*—H···*A*	*D*—H	H···*A*	*D*···*A*	*D*—H···*A*
O3*A*—H3*A*···O1*A*^i^	0.87 (2)	1.84 (2)	2.6878 (15)	164.9 (17)
O3*A*—H3*A*···O2*A*^i^	0.87 (2)	2.52 (2)	3.1629 (14)	130.6 (16)
N1*B*—H1*BA*···O2*A*^i^	0.85 (2)	1.90 (2)	2.7457 (16)	176.1 (16)
N1*B*—H1*BB*···O1*A*	0.98 (2)	1.78 (2)	2.7337 (15)	163.7 (18)
N1*B*—H1*BB*···O3*A*	0.98 (2)	2.42 (2)	3.0019 (14)	117.4 (15)
C6*B*—H6*B*···O2*A*^i^	0.92 (2)	2.39 (2)	3.2275 (19)	152.1 (15)
C2*A*—H2*A*···*Cg*2	1.00 (2)	2.827 (19)	3.7029 (18)	146.7 (14)
C9*B*—H9*B*2···*Cg*2^ii^	0.97 (2)	2.69 (2)	3.4243 (17)	146.7 (14)
C7*A*—H7*A*···*Cg*1^iii^	0.99 (2)	2.753 (19)	3.6111 (18)	145.5 (15)

Symmetry codes: (i) −*x*+3/2, *y*−1/2, −*z*+1; (ii) −*x*+2, *y*, −*z*+1; (iii) *x*, *y*, *z*+1.
