# Supplementary figures and images for: Crystal structure of (R)-N-benzyl-1-phenylethanaminium (R)-4-chloro­mandelate
Source: Acta Crystallogr Sect E Struct Rep Online. 2014 Nov 5;70(Pt 12):o1223–4. doi: 10.1107/S1600536814023204 (PMC4257388; doi:10.1107/S1600536814023204)

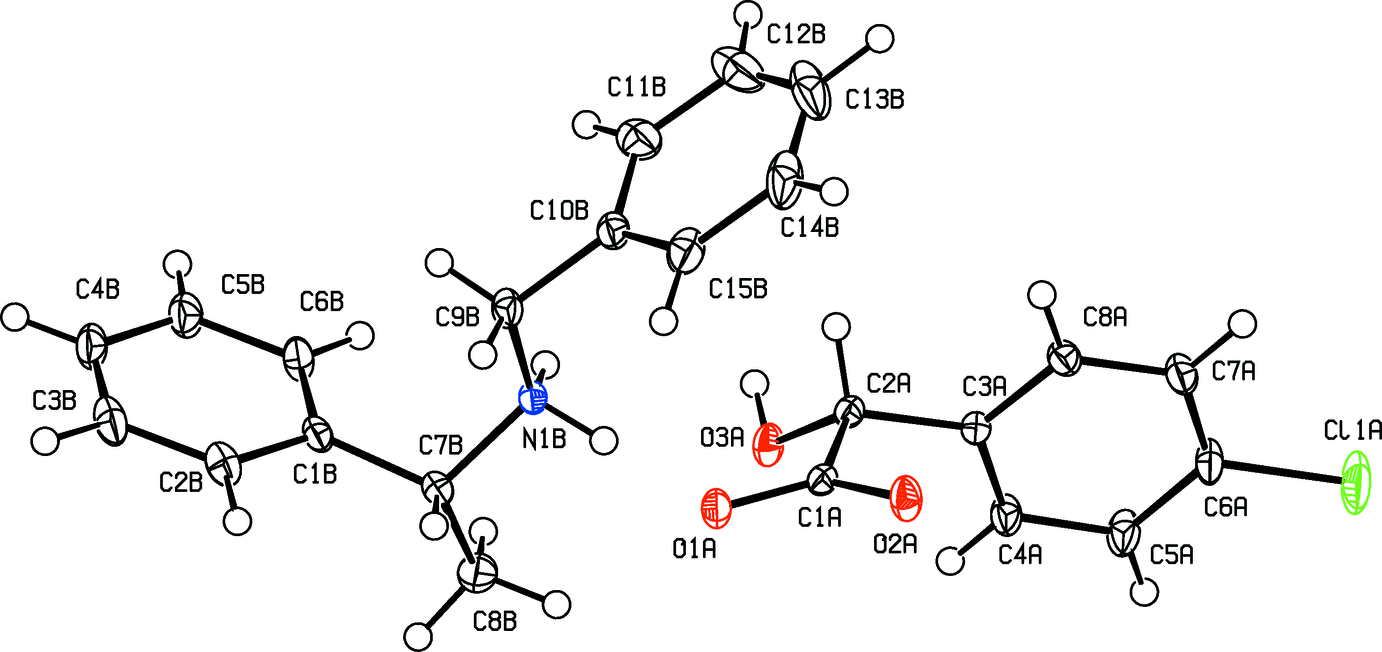

Supplement: Supplementary file 3 [file e-70-o1223-fig1.tif]

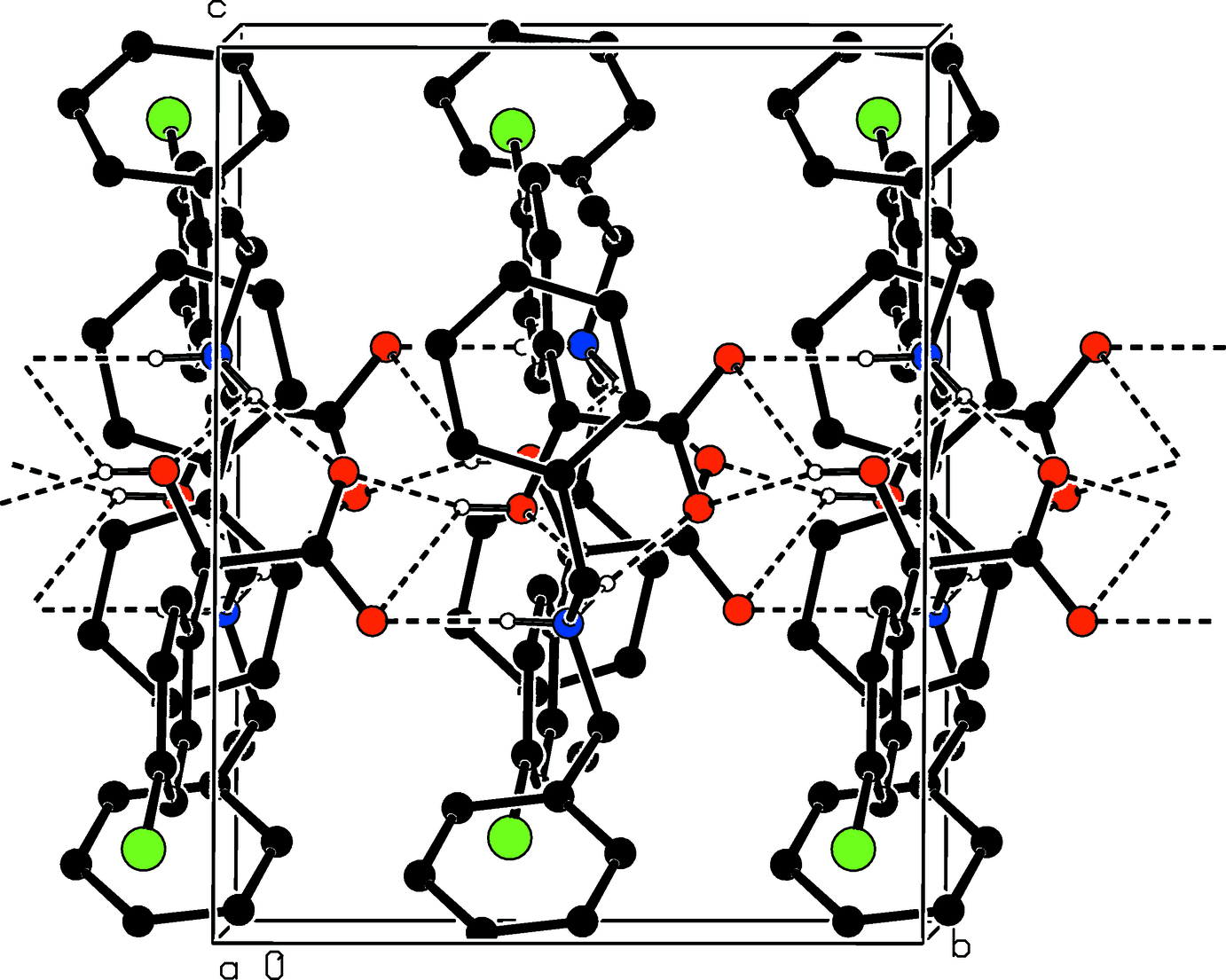

Supplement: Supplementary file 4 [file e-70-o1223-fig2.tif]
